# A multifactorial analysis of obesity as CVD risk factor: Use of neural network based methods in a nutrigenetics context

**DOI:** 10.1186/1471-2105-11-453

**Published:** 2010-09-08

**Authors:** Ioannis K Valavanis, Stavroula G Mougiakakou, Keith A Grimaldi, Konstantina S Nikita

**Affiliations:** 1 School of Electrical and Computer Engineering, National Technical University of Athens, 9 Iroon Polytechniou Str., 15780, Zografos, Athens, Greece; 2 University of Bern, Medical School, ARTORG Center for Biomedical Engineering Research, Stauffacherstrasse 78, 3014 Bern, Switzerland; 3 University Hospital - Inselspital - University of Bern, Division of Endocrinology, Diabetes and Clinical Nutrition, 3010 Bern, Switzerland; 4 Sciona, Inc., 12635 E. Montview Blvd. #217, Aurora, CO 80045-7337, USA

## Abstract

**Background:**

Obesity is a multifactorial trait, which comprises an independent risk factor for cardiovascular disease (CVD). The aim of the current work is to study the complex etiology beneath obesity and identify genetic variations and/or factors related to nutrition that contribute to its variability. To this end, a set of more than 2300 white subjects who participated in a nutrigenetics study was used. For each subject a total of 63 factors describing genetic variants related to CVD (24 in total), gender, and nutrition (38 in total), e.g. average daily intake in calories and cholesterol, were measured. Each subject was categorized according to body mass index (BMI) as normal (BMI ≤ 25) or overweight (BMI > 25). Two artificial neural network (ANN) based methods were designed and used towards the analysis of the available data. These corresponded to i) a multi-layer feed-forward ANN combined with a parameter decreasing method (PDM-ANN), and ii) a multi-layer feed-forward ANN trained by a hybrid method (GA-ANN) which combines genetic algorithms and the popular back-propagation training algorithm.

**Results:**

PDM-ANN and GA-ANN were comparatively assessed in terms of their ability to identify the most important factors among the initial 63 variables describing genetic variations, nutrition and gender, able to classify a subject into one of the BMI related classes: normal and overweight. The methods were designed and evaluated using appropriate training and testing sets provided by 3-fold Cross Validation (3-CV) resampling. Classification accuracy, sensitivity, specificity and area under receiver operating characteristics curve were utilized to evaluate the resulted predictive ANN models. The most parsimonious set of factors was obtained by the GA-ANN method and included gender, six genetic variations and 18 nutrition-related variables. The corresponding predictive model was characterized by a mean accuracy equal of 61.46% in the 3-CV testing sets.

**Conclusions:**

The ANN based methods revealed factors that interactively contribute to obesity trait and provided predictive models with a promising generalization ability. In general, results showed that ANNs and their hybrids can provide useful tools for the study of complex traits in the context of nutrigenetics.

## Background

Cardiovascular disease (CVD) is a family of common multifactorial diseases, e.g. coronary heart disease (CHD), cerebrovascular disease, hypertension, and heart failure, which develop as a consequence of interactions between the "initial" conditions, coded in a person's genotype, and exposure to environmental factors (e.g. nutrition, smoking) [1]. Latest statistics shows that CVDs are the leading cause of death and morbidity worldwide and according to the World Health Organization (WHO) an estimated 16.7 million - or 29.2% of deaths - result from the various forms of CVD. However, many CVDs are preventable by action on the primary environmental risk factors such as unhealthy diet, physical inactivity, and smoking [2]. Obesity comprises one of the most important independent CVD risk factors and many large scale studies have shown a positive relationship between CVD mortality and body mass index (BMI), a widely used measure of human obesity [3-6]. Nutritional changes towards westernized diet, high in sugar and fats, and the sedentary lifestyle have led to increased obesity and CVD prevalence even in the developing countries [7-9]. Although interventions on a person's nutrition can reduce BMI, it has been shown that efforts towards BMI reduction can be affected by a person's genetic profile [10]. The synergy of genes and nutrition is studied within the new fields of nutrigenetics and nutrigenomics [11]. These new disciplines establish new strategies for CVD control which traditionally has been limited to nutrition interventions (e.g. fruits, vegetables, fish) and supplementation, the latter being more popular in American population [12].

In order to reveal how genes and environmental factors, like nutrition, interact to perturb biological pathways that cause multifactorial diseases, advanced computational methods able to indentify inter- and intra-correlation on diverse sources of information can be applied [13]. The methods usually applied in literature aim to identify gene-gene and/or gene-environment interactions that contribute to the onset of a disease, and develop predictive models which can assess a person's risk to be affected by the disease. A novel diagnostic prediction method for allergic diseases (atopic dermatitis, allergic conjunctivitis, allergic rhinitis and bronchial asthma) that used SNP data and an artificial neural network (ANN) architecture was proposed in [14], resulting in a diagnostic prediction accuracy equal to 78%. An ANN combined with a parameter decreasing method (PDM-ANN) was utilized to analyze 25 SNPs from 17 genes and select the most informative SNPs combination related to childhood allergic asthma in [15]. Ten SNPs were identified as the most informative and were used by the ANN predictive model that yielded an accuracy of 74.4%. An ANN optimized by genetic programming (GP) [16] was used for the study of Parkinson's disease, revealing a strong correlation between the *DSTL *gene and gender with the disease [17]. Random forest is a collection of classification trees able to build a high-dimensional non-parametric predictive model and was applied to study associations between asthma and various SNPs of *ADAM33 *gene [18]. Multifactorial dimensionality reduction (MDR) is a popular combinatorial method that uses a constructive induction algorithm to convert two or more factors to a single attribute and was successfully applied for the detection of multi-locus interactions in prostate cancer [19], breast cancer [20,21], and type 2 diabetes [22]. Finally, support vector machines (SVMs) were used towards personalized risk assessment of type 1 diabetes using SNP genotype data [23]. Research conducted for revealing gene-environment interactions towards a disease trait is to our knowledge rather less than research aiming to reveal gene-gene interactions. This can be due to the lack of data on appropriate environmental factors describing subjects and the high complexity that can characterize such a study, i.e. a vast number of categorical/continuous variables to analyze that require an extensive subjects' sample. Related research efforts estimated the strength of associations of multiple SNPs and environmental factors with diabetes using a combination of a logistic regression (LR) model and genetic algorithm (GA) [24], and support vector machines [25], while a Bayesian mixture model was proposed for modelling gene-environment interactions in the study of lung cancer in [26].

Regarding the complex etiology of CVD, the MDR approach was used in order to study gene-gene interactions on the onset of hypertension [27]. The study showed that two genes, *ACE *and *GRK4*, affecting blood pressure are involved in hypertension onset and the corresponding genotype was sufficient to predict the disease's phenotype with an accuracy of 70.5%. The CVD risk factor of obesity, in particular, was studied using monozygotic/dizygotic twin pairs and statistical analysis [28-30]. It was shown that BMI is characterized by both genetic susceptibility (without, though reporting specific genes) and environmental factors, e.g. fiber intake and physical activity [30]. Finally, statistical analysis in [31] concluded that *Pro12Ala *polymorphism in peroxisome proliferator-activated receptor and *ghrelin Leu72Met *polymorphism affect interactively with dietary fat the modulated waist circumference, which measures human weight.

The aim of the present paper is to study the etiology of obesity as an example of CVD risk factor and identify its association with gender, genetic variations and nutrition habits. To this end, a set of more than 2300 white people that have participated in a nutrigenetics test were used and two ANN approaches were applied. Obesity was measured here using BMI, while nutrition habits were quantified using daily intake through food and supplements of various substances, e.g. cholesterol and saturated fat. Genetic variations came from a panel of 24 SNPs or Insertions/Deletions that are related to human weight and cardiovascular health aspects. The selected genetic variations have all been reported to interact in some way with nutritional components, modifying the daily requirements for various micro and macro-nutrients and affecting upper recommended limits of potentially harmful components such as saturated fats [10]. The multifactorial analysis of obesity was approached using ANN based methods for the classification of subjects into two classes related to human weight: normal (BMI ≤ 25) and overweight (BMI > 25). More specifically, PDM-ANN [13,15] and GA-ANN, were used. PDM-ANN combines a backwards feature selection method and an ANN in order to define the most informative subset of factors. GA-ANN is an in-house inspired and developed algorithm that permits automatic selection of i) the most important input variables and ii) the ANN architecture and training process parameters, towards optimization of the ANN performance in terms of classification accuracy. The GA-ANN has been successfully used in various applications towards landscape classification [32] and diagnosis of carotid atherosclerosis from ultrasound images [33]. We decided to approach the multifactorial trait of obesity using ANN-based methods rather than simple statistics, e.g. X^2 ^independence test or analysis of variance (ANOVA), aiming at revealing high dimensionality interactions and constructing prediction models, since in similar studies with smaller datasets the PDM-ANN outperformed the X^2 ^and ANOVA methods [34]. The currnet work is to authors' knowledge the first that processes data which describe subjects' genes and nutrition using computational intelligence techniques, and shows the importance of ANNs and their hybrids in the new field of nutrigenetics in the post-genomic era.

## Dataset

A set of 2341 white people that underwent a nutrigenetic test was used. The test included the collection of genotype and nutrient intake data for all subjects along with the BMI measurement. The Sciona MyCellf™ kit (Sciona Inc., Boulder, CO, USA) was used in order to acquire data related to subjects' genotype and nutrition. More specifically, all subjects completed a comprehensive diet and lifestyle questionnaire, while cheek cell samples were taken for genetic testing purposes. Nutrient intake measurements were determined from the responses to the diet questionnaire and depicted their average dietary habits. A total of 38 nutrition related measurements were calculated. These included the total calories intake per day and the daily intake, through food or supplements, for various substances, i.e. calcium, allium, caffeine, cruciferous, folic acid, cholesterol, omega 3, refined carbohydrate, saturated fat, and vitamins A, B_6_, B_12_, C, D, E. For the substances that were taken as supplements by at least one subject (e.g. calcium and vitamins), two other measurements: "intake in supplement" and "total intake"- were calculated apart from "intake in food". Nutrition intake measurements were categorized into four classes. To this end, for "intake in food" and "total intake" measurements the quartiles were found, while for the "intake in supplement" measurement, the first class corresponded to a zero intake and the remaining classes corresponded to the bottom 33.3%, middle 33.3% and top 33.3% of non-zero values. The cheek cell samples underwent genetic testing using a Sequenom Mass Array system for 24 genetic variations (SNPs or Insertion(I)/Deletion(D)) related to nutrition and CVD risk. The genetic variations set included *ACE I/D*, *APOC3 C3175G*, *CBS C699T*, *CETP G279A*, *COL1A1 G Sp1 T*, *GSTM1 deletion*, *GSTP1 A313G*, *GSTP1 C341T*, *GSTT1 deletion*, *IL 6 G634C*, *IL 6 G174C*, *LPL 1595G*, *MTHFR C677T*, *MTHFR A1298C*, *MTR A2756G*, *MS MTRR A66G*, *NOS3 G894T*, *PPAR gamma 2 Pro12Ala*, *SOD2 C28T*, *SOD3 C760G*, *TNF alpha G308A*, *VDR Fok1*, *VDR Bsm1 *and *VDR Taq1 *[10]. Each genetic variation was featured one out of three forms (e.g. AA, GG and AG for the *CETP G279A *SNP, and II, DD, ID for the *ACE I/D *variation), resulting to a three-class variable. For each subject, the gender was also known and was used as a two-class variable (male/female). Finally, BMI, i.e. weight (Kg)/height^2 ^(m^2^), was calculated for all subjects: 877 out of 2341 were characterized as normal (BMI ≤ 25), while the rest 1464 subjects as overweight (BMI > 25). The subjects comprised customers of the Sciona service in the USA and had provided signed consent to genetic testing and the anonymous use of their data for research purposes. The chosen subjects were a mixture of male (910 subjects or 38.9%) and female (1431 subjects or 61.1%), all self-declared white ethinicity. Their age was in the range of 20-78 (median = 51, mean ± std = 50.56 ± 11.80) projected to the following age groups: 20-35:10.9%, 36-50: 36.6%, 51-65:43.1% and >65: 9.4%. In order to justify the use of the ANNs, able to capture complex relationships within data, we calculated in a pre-processing step the linear coefficient (LC) values between each nutrition intake measurement and BMI in their continuous form. Only the measurements of total cholesterol intake (LC = 10.84), cholesterol intake in food (LC = 11.03), refined carbohydrate intake in food (LC = 10.56), total saturated fat intake (LC = 16.33) and saturated fat intake in food (LC = 16.45) featured an LC with an absolute value greater than 0.1, showing a very weak linear relationship.

## Methods

PDM-ANN and GA-ANN were applied in order to investigate the relation of the 63 factors that comprised the categorical factors describing genotype (24), nutrition habits (38) and gender, to BMI measurement. The 63 factors were fed as input variables to the ANN methods, while the BMI measurement was used as a categorical two class output variable. Both methods simultaneously performed feature selection, i.e. they selected the most important factors that affect the output variable, and constructed predictive models.

### PDM-ANN

PDM-ANN is based on the combined use of a backward feature selection method and an ANN [13,15]. Initially, the full vector of input factors is applied as input to the ANN and PDM eliminates serially the factors that are less associated with the ANN output. The procedure is repeated until one factor remains in the feature vector. In the present study, a feed-forward ANN [35] was used, consisting of one input layer of input neurons equal in number to the applied factors, one hidden layer of adjustable number of neurons, and one output layer of one neuron. The hyperbolic tangent sigmoid and log sigmoid were used as activation functions in the hidden and output layer, respectively. Due to the use of hyperbolic tangent sigmoid function, the labels of categorical input variables were set to certain values in the range [-1.0, +1.0], e.g. the labels [1^st^, 2^nd^, 3^rd ^and 4^th ^class] of a 4-class variable were set to [-1.000, -0.333, +0.333, +1.000].The use of log sigmoid function in the output neuron resulted in output values in the range [0.0, +1.0]. A value of less than +0.5 corresponded to BMI ≤ 25 (C1), while a value greater or equal to +0.5 corresponded to BMI > 25 (C2). ANN was trained using the back-propagation algorithm with adaptive learning rate and momentum in order to control the ANN training procedure in terms of convergence rate [35]. Initial learning rate (lr) and momentum (mc) were set to lr = 0.01 and mc = 0.9, respectively, while several values for the number of hidden neurons (number of hidden neurons = 1, 2, 4, 6 and 8) were tested.

The PDM-ANN process is described in detail in the following: It starts by constructing an ANN that uses all *N *= 63 input factors. For proper ANN training and testing the available data have been split into training and testing sets following the 3- fold Cross Validation (3-CV) resampling [36]. Training and testing is, thus, repeated three times. Each time the ANN is trained using 2/3 (~67%) randomly chosen cases of the available dataset and tested using the remaining cases (~33%). Each of the ANNs obtained by 3-CV (ANN_1_, ANN_2_, ANN_3_) is evaluated, similarly as done in [15], using the mean value of the classification accuracies $\left({\bar{A}}_{1(N)},{\bar{A}}_{2(N)},{\bar{A}}_{3(N)}\right)$ obtained in the corresponding 3-CV training and testing sets. It is noted that the accuracy achieved by an ANN in a set is the fraction of cases that are correctly classified by the ANN. The 3-CV technique outputs a fitness value (%):

(1) $$F_{N}=({\bar{A}}_{1(N)}+{\bar{A}}_{2(N)}+{\bar{A}}_{3(N)})/3$$

for the initial ANNs that use all *N *= 63 input factors. The procedure continues by deleting one factor from the total number of factors and constructing the 3-CV ANNs that use the remaining factors as input. In turn, each factor is deleted from the total number of factors and ANNs are constructed with the remaining ones. The 62-dimensional input factors set that yields the best average of mean accuracies $\left({\bar{A}}_{1(62)},{\bar{A}}_{2(62)},{\bar{A}}_{3(62)}\right)$ in the 3-CV sets is assigned the fitness value *F*_*N = 62 *_(eq(1)) and is the one chosen at this dimensionality threshold. As the procedure goes on, an ANN that uses *N *inputs is derived from the one that uses *N *+ 1 inputs by subtracting the least informative factor by means of mean accuracy in the 3-CV training and testing sets, and the set of *N *factors is assigned a fitness value *F_N _*. This process is repeated until one factor remains. The best set of 3-CV ANNs and the corresponding input factors set are selected based on the values of fitness function *F*. The factors that are included in the selected set of factors are the most informative ones, while the ones left out are either redundant or do not affect the output.

### GA-ANN

GA-ANN combines the evolutionary optimization method of GA [37] with an ANN classifier in order to obtain an optimal ANN architecture [38]. A three layer feed-forward ANN was again used, trained by the back-propagation algorithm with adaptive learning rate and momentum. The hyperbolic tangent sigmoid and log sigmoid were used as activation functions in the hidden and output layer, respectively, and the input values were used after their encoding in the range [-1.0, 1.0] as described for the PDM-ANN method. The GA was applied in order to optimize the ANN architecture, including the set of input factors to be used, and the fitness function, used by PDM-ANN, was utilized.

The whole GA-ANN process is the following: An initial generation of *M *= 100 random chromosomes is created within the first step of the GA. Each chromosome is a binary mask of *lchrom *= 76 binary digits (0 or 1). The *lchrom *digits of each chromosome encode the architecture of one ANN: i) the factors to be used as input in the training procedure are encoded by the first *lf *= 63 digits, ii) the number of hidden neurons by the next *lh *= 4 digits, iii) the range of the initial weights by the next *lw *= 3 digits, iv) the momentum term by the next *lm *= 4 bits, and v) the initial learning rate by the last *ll *= 2 bits. Three ANNs are constructed for each of the *M *chromosomes according to its content and tested within the 3-CV technique. The corresponding classification accuracies in 3-CV training and testing sets are measured and the fitness function value of each chromosome is calculated. Four genetic operators, i.e. selection, crossover, mutation, and election, are then applied to the initial generation of chromosomes. The selection operator uses the elitist selection method [37] and selects the chromosomes that will mate to produce the offsprings for the next generation. Random pairs of the selected chromosomes mate with probability *P_c _*= 0.7 based on the two-point crossover operator [37] and bits within a chromosome are mutated (switched from 0 to 1 or vice-versa) with probability *P_m _*= 0.01. In order to avoid that offsprings with a lower fitness value than their parents are included in the next generation, the election operator is finally used [38]. For the sake of low dimensionality of the selected subset of input factors, "penalty" function is applied to chromosomes that encode a subset exceeding a given dimensionality threshold *T*. Thus, these chromosomes are assigned a fitness value equal to 50% of the average population fitness. The whole procedure is repeated for *N_G _*= 50 generations and results are stored. The algorithm yields to the optimal ANN classifier, which corresponds to the chromosome featured the maximum fitness value.

The GA-ANN method was applied for dimensionality thresholds *T *= 30 and *T *= *Inf*, the latter corresponding to the case where no penalty function is applied and the method searches for the optimal set of factors with no limitation in terms of dimensionality.

The finally selected predictive models of PDM-ANN and GA-ANN were comparatively assessed using the metrics of accuracy as well as, sensitivity, i.e. the discriminative power regarding positive cases (BMI > 25), specificity, i.e. the discriminative power regarding negative cases (BMI ≤ 25), and area under Receiver Operating Characteristics (ROC) curve. ROC curves were generated by thresholding the output neuron in the range [0, 1], with a step equal to 0.02, and estimating the true positive (sensitivity) and false positive (1-specificity) rates for each threshold. All metrics were calculated for all 3-CV sets and mean values were computed separately for the training and testing sets.

Furthermore, a permutation testing methodology [39,40] was utilized in order to ascribe statistical significance to all evaluation measurements (accuracy, sensitivity, specificity, area under ROC curve) obtained for each of the resulting models in the 3-CV testing sets. The statistical significance *p *of the value obtained for an evaluation measurement Q by a predictive model was calculated as follows: The output class labels of all cases in a 3-CV testing set were S(= 1000) times randomly permuted. The value of Q was calculated for each of the S sets, providing the distribution of Q under the null hypothesis of no association between the input factors fed to the ANN and the output. The number of times any of these values exceeded the value of Q obtained for the real testing set was denoted with R. The quantity

(2) p = (R+1) / (S+1)

provides an unbiased estimate of the statistical significance of the obtained value of Q in the 3-CV testing set. The whole procedure was repeated for each 3-CV testing set and the mean value of *p *was calculated.

## Results and Discussion

In this section, the results of PDM-ANN method are firstly reported followed by these obtained by GA-ANN. Finally, both methods are comparatively assessed and results are discussed.

### PDM-ANN Results

**Figure 1:**
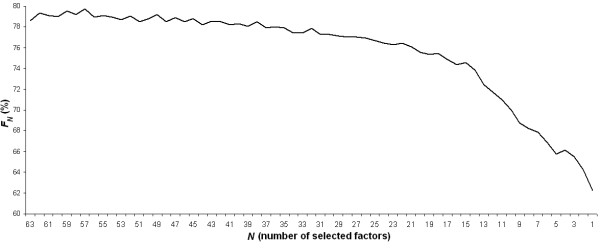
**Fitness (*F_N _*) values obtained per number of selected factors (*N*) using the PDM-ANN method**.

The PDM-ANN method was firstly applied in order to identify the most important factors among the aforementioned genetic variations, nutrient intake measurements and gender that affect BMI when used as a two class output variable (C1 vs. C2). The obtained *F_N _*values per number of selected factors *N *= 63, 62, ..., 1 are presented in Figure 1. Results show that the mean accuracy obtained in the 3-CV training and testing sets when using *N *factors was kept in the range 77.7%-79.6% for *N *= 63,..,32 and it started to decrease for *N *< 32. It continued to decrease up to *N *= 1, where a mean accuracy equal to 62.3% was obtained. The fact that the factors subtracted by the PDM up to the threshold *N *= 32 did not affect the *F_N _*values indicates that these certain factors either do not have an important impact to BMI measurement or are redundant with other factors kept in the input variables set. The one by one subtraction of factors after dimensionality *N *= 32 caused a progressive reduction to *F_N _*values and it is inferred that the factors subtracted after this dimensionality threshold have a high information content towards BMI measurement. Thus, the subset of 32 factors was considered as optimal for the classification of subjects according to BMI measurement in terms of obtained mean accuracy and number of used factors.

Table 1 presents the *F_N _*values and mean accuracies in the 3-CV training and testing sets per number of selected variables *N *= 1, 2, 3, 4, 5 and 30, 31, 32 (the in-between cases are not reported here for room saving purposes). *F_N _*values show the decrease of the mean accuracy obtained in 3-CV sets as the dimensionality N ≤ 32 reduces, while the same happened for accuracies obtained in the training sets. The selected 32-dimensional subset of factors is presented in the bottom of Table 1 and contained gender, 19 nutrition related factors e.g. calories and cholesterol with an obvious impact on a subject's BMI, and twelve genetic variations. Thus, the final subset of factors selected by PDM-ANN contained factors reflecting subject's gender, lifestyle, i.e. nutrition, and genetic profile. These interact with each other (*n*-way interactions, *n *> 1) towards the complex trait of BMI or act as factors that contribute solely to the BMI trait (main interactions). It is important to note here that even when training the ANNs with one variable, i.e. the Cholesterol-Intake in Food, a high *F*_*N = 1 *_value (~62%) was obtained, as well. This can be explained by the effect that the intake of cholesterol can have on a subject's BMI, and by that a high intake of cholesterol is usually accompanied by a high intake of saturated fats and calories. The ANN-based predictive model that used the 32 selected factors as input achieved a mean accuracy equal to 77.89% in the 3-CV training and testing sets. Regarding its ability to generalize into totally unknown data, the corresponding mean accuracy, achieved in the 3-CV testing sets, was 60.22%.

**Table 1:** Selected factors, *F_N _*values and mean accuracies in the 3-CV training and testing sets obtained by PDM-ANN for dimensionality *N *= 1,..5,30-32.

Dimensionality *N*	Selected Factors	*F_N _*(%)	Mean Training Accuracy (%)	Mean Testing Accuracy (%)
1	Cholesterol-Intake in Food	62.23	63.16	61.29

2	(*N *= 1) + Gender	64.24	64.17	64.32

3	(*N *= 2) + Vitamin A-Total Intake	65.52	65.71	65.34

4	(*N *= 3) + Omega 3-Intake in Supplement	66.12	67.37	64.87

5	(*N *= 4) + *VDR Fok1*	65.75	68.85	62.66

..	....	....	....	....

30	(*N *= 31) -Vitamin B_12_-Intake in Food	77.29	94.74	59.83

31	(*N *= 32) - TNF alpha *G308A*	77.30	94.21	60.39

32	Gender, Calories, Calcium-Intake in Food, Calcium-Supplement Only, Allium- Intake in Food, Folic Acid-Supplement, Cholesterol-Intake in Food, Cholesterol-Intake in Supplement, Omega 3-Intake in Food, Omega 3-Intake in Supplement, Saturated Fat-Intake in Supplement, Vitamin A-Total Intake, Vitamin A-Intake in Food, Vitamin A-Intake in Supplement, Vitamin B_6_-Total Intake, Vitamin B_6_-Intake in Food, Vitamin B_6_-Intake in Supplement, Vitamin B_12_-Total Intake, Vitamin B_12_-Intake in Food, Vitamin C-Total Intake, *CBS C699T*, *CETP G279A*, *COL1A1 G Sp1 T GSTM1 deletion, GSTP1 A313G, GSTT1 deletion, IL 6 G634C, MTHFR C677T, SOD2 C28T, TNF alpha G308A, VDR Fok1*, *VDR Bsm1 *(32)	77.89	95.56	60.22

### GA-ANN Results

**Figure 2:**
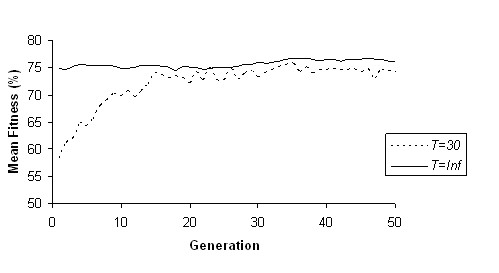
**Mean fitness value of chromosomes in each generation during GA evolution within the GA-ANN method (*T *= *Inf *and *T *= 30)**.

GA-ANN was next applied in order to construct an optimal ANN in terms of input factors, architecture and training parameters (number of hidden neurons, momentum and learning rate of the ANN), which leads to a maximum classification accuracy. The procedure was followed both for *T *= *Inf *and *T *= 30 and results for the fitness value and mean accuracies in 3-CV training and testing sets were stored while GA evolved. Figure 2 presents the mean fitness value of all chromosomes obtained in each generation. Results show that while GA evolved, the fitness function improved in average and thus better ANN architectures for discriminating subjects in terms of BMI were obtained. This is more clear in the case *T *= 30 where the mean fitness value improved from 58.4% to 74.24%. After GA-ANN run for 50 generations, the ANN that corresponds to the chromosome of the best fitness value was selected as the optimal one. When no dimensionality threshold was applied (*T *= *Inf*) the best ANN (fitness = 79.18%) was obtained during the 47^th ^generation and used 32 out of 63 factors as inputs. In case of dimensionality threshold *T *= 30, the best ANN (fitness = 79.38%) was obtained again during the 47^th ^generation and was fed by a selected subset of 25 factors. Thus, the application of dimensionality threshold led to an input set of lower dimensionality, as compared to the case of no dimensionality threshold, provided though the same high fitness value. Table 2 presents the subsets of factors fed as inputs to the best ANNs for *T *= *Inf *and *T *= 30. The selected subsets included gender and factors related to nutrition and genetic variations, thus showing the multifactorial contribution of the studied factors to BMI.

**Table 2:** Sets of factors fed as inputs to the optimal ANNs obtained by GA-ANN (*T *= *Inf *and *T *= 30)

*T = Inf*	*T *= 30
Gender, Calcium- Total Intake,	Gender, Calories, Calcium-
Calcium- Intake in Food, Allium-	Total Intake, Allium- Intake in
Intake in Food, Cruciferous-Intake in	Food, Caffeine-Total Intake,
Food, Folic Acid- Intake in Food,	Folic Acid-Intake in
Folic Acid- Intake in Supplement,	Supplement, Cholesterol-
Cholesterol-Intake in Food,	Intake in Food, Cholesterol-
Cholesterol-Intake in Supplement,	Intake in Supplement, Omega
Omega 3-Total Intake, Omega 3-	3-Total Intake, Omega 3-
Intake in Food, Omega 3-Intake in	Intake in Food, Refined
Supplement, Vitamin A-Total Intake,	Carbohydrate- Intake in Food,
Vitamin A-Intake in Food, Vitamin	Saturated Fat-Intake in Food,
B_6_-Total Intake, Vitamin B_6_-Intake in	Vitamin A-Intake in
Food, Vitamin B_12_-Total Intake,	Supplement, Vitamin B_12_-
Vitamin B_12_-Intake in Supplement,	Intake in Supplement, Vitamin
Vitamin C-Intake in Supplement,	C-Total Intake, Vitamin C-
Vitamin D-Total Intake, Vitamin D-	Intake in Supplement, Vitamin
Intake in Food, Vitamin D-Intake in	D-Total Intake, Vitamin D-
Supplement, Vitamin E-Total Intake,	Intake in Food, Vitamin E-
Vitamin E-Intake in Supplement, *CBS*	Food Only, *IL 6 G174C*, *LPL*
*C699T, COL1A1 G Sp1 T, GSTP1*	*1595G, MTHFR C677T*,
*C341T, LPL 1595G, MTHFR C677T*,	*MTHFR A1298C*, *MTR*
*MTHFR A1298C, MTR A2756G*,	*A2756G*, *PPAR gamma 2*
*NOS3 G894T *(32 factors in total)	*Pro12Ala *(25 factors in total)

### Comparative Assessment and Discussion

The mean values of accuracy, sensitivity, specificity and area under ROC curve obtained in the 3-CV training and testing sets by i) PDM-ANN (32 factors), ii) GA-ANN (*T *= *Inf*) (32 factors), and iii) GA-ANN (*T *= 30) (25 factors), are presented in Table 3. Regarding performance in the 3-CV testing sets, all finally selected ANN architectures provided mean accuracies in the range 60%-62%, which can be considered quite satisfying. It is important though to note that the trained ANNs provided a quite higher true positive rate (mean sensitivity in the range 69%-71%) and a lower true negative rate (mean specificity in the range 44%-49%). This is a desirable result since the aim of the constructed ANN-based system is to predict a subject's future BMI status using information on his/her gender, nutrition habits and genetic profile. Thus, it is more important for the system to be able to predict a high BMI, characterized as an independent risk factor for CVD, than to predict a low BMI. The mean statistical significance of the obtained values of all evaluation measurements (accuracy, sensitivity, specificity, and area under ROC curve) in the 3-CV testing sets were found very high (*p *< 0.001) using the permutation testing methodology in the context of expectation under the null hypothesis of no association. To be more exact, the evaluation measurements in all S(= 1000) randomly permuted sets were found less than the value obtained in the 3-CV testing sets, i.e. R = 0, and all *p*-values were found equal to *p *= (0+1)/(1000+1) < 0.001 (see eq. (2) in Methods section).

**Table 3:** Mean Accuracy, Sensitivity, Specificity and Area under ROC curve in the 3-CV sets for the ANN architectures obtained by the PDM-ANN and the GA-ANN (*T *= *Inf *and *T *= 30)

Measurement	ANN architecture	Mean Value in 3-CV Training Sets	Mean Value in 3-CV Testing Sets
	PDM-ANN (32 factors)	95.56	60.22
	
Accuracy (%)	GA-ANN, *T *= *Inf* (32 factors)	97.67	60.69
	
	GA-ANN, *T *= 30 (25 factors)	97.10	61.46

	PDM-ANN (32 factors)	98.14	69.15
	
Sensitivity (%)	GA-ANN, *T = Inf* (32 factors)	99.39	70.79
	
	GA-ANN, *T *= 30 (25 factors)	98.90	69.80

	PDM-ANN (32 factors)	91.15	46.08
	
Specificity (%)	GA-ANN, *T = Inf* (32 factors)	94.73	44.62
	
	GA-ANN, *T *= 30 (25 factors)	94.54	48.63

	PDM-ANN (32 factors)	0.941	0.580
	
Area under ROC curve	GA-ANN, *T *= *Inf* (32 factors)	0.969	0.574
	
	GA-ANN, *T *= 30 (25 factors)	0.964	0.608

The ANN obtained by GA-ANN (*T *= 30) was the best performing architecture in the 3-CV testing sets in terms of mean accuracy (61.46%), mean specificity (48.63%), mean area under ROC curve (0.608), and second best performing in terms of mean sensitivity (69.80%) after the one obtained by GA-ANN (*T *= *Inf*). It was also in favor of the specific architecture that it was fed by the most parsimonious set of factors obtained by the applied methods with a dimensionality equal to 25. It is, thus, shown here that the stochastic feature selection within the GA-ANN yielded better results than the serial backward feature selection within PDM-ANN.

For comparison reasons we used the linear discrimination analysis (LDA) method using the 38 continuous nutrition intake measurements in order to predict the status of the two class BMI output variable. Only continuous input variables can feed LDA and the factors corresponding to gender and genetic variations, available only as categorical variables, were discarded. The method provided mean values of accuracy, sensitivity and specificity equal to 58.2%, 58.3% and 58.1%, respectively, in the 3-CV testing sets and was outperformed by the selected models of PDM-ANN and GA-ANN in terms of accuracy and sensitivity. This is due to that PDM-ANN and GA-ANN can manipulate all factors (gender, nutrition measurements and genetic variations) after their transformation to categorical input factors, and simultaneously capture non-linear relationships. Even when LDA was fed with the sole factor selected by PDM-ANN (*N *= 1), i.e. cholesterol intake in food, it was outperformed by the corresponding PDM-ANN model in terms of mean accuracy (52.7% versus 60.2%).

It is important to discuss the factor selection results obtained here by the ANN-based methods. The studied factors may have a similar impact on the BMI trait, e.g. calories and cholesterol intake measurements, or intake of a vitamin in food and intake of a vitamin in supplement, and the selected ones may depend on the selection method used. Thus, the applied methods (PDM-ANN, GA-ANN (*T *= *Inf*), GA-ANN (*T *= 30) resulted to three overlapping subsets of factors suggested to contribute to BMI. Moreover, the selected subsets include common factors, e.g. the genetic variation of *MTHFR C677T*, Gender and Cholesterol-Intake in Food were suggested by all methods, while the genetic variations of *CBS C699T*, *MTR A2756G *and calories measurement were suggested by two out of three methods (*CBS C699T *by PDM-ANN and GA-ANN (*T *= *Inf*), MTR A2756G by GA-ANN (*T *= *Inf *and *T *= 30) and calories by PDM-ANN and GA-ANN (*T *= 30)). The consistency of these factors shows their strong impact. Nutrition related factors like calories and cholesterol intake have an obvious impact on BMI and are well known from daily life to trigger the onset of obesity. However, the current study showed the existence of other factors, i.e. intake of vitamins and genetic variations that complete the optimal sets of factors that can discriminate subjects with high or low BMI. It is worth noting that the polymorphisms of *PPAR gamma 2 Pro12Ala*, selected by GA-ANN (*T *= 30), and *TNF alpha G308A*, selected by PDM-ANN, have been related to obesity in [41] and [42], as well, respectively. Further research on the biochemical pathways in which the selected genes are involved could enlighten the way they solely affect BMI or how they interact with nutrition towards the complex BMI trait. Related examples include that the recommended intake of folic acid required to keep homocysteine (independent CVD risk factor) levels normal depends on the *MTHFR *gene variation, while the upper limits of saturated fat intake depend on the *CETP*, *LPL *and *APOC3 *genes.

The optimal ANN architecture yielded by GA-ANN has been integrated with a remotely located rule-based module that provides personalized information on required modifications of a subject's lifestyle habits in order to reduce the risk of complications related to the cardiovascular system. The proposed modifications are based on existing knowledge in the literature, regarding mostly the combined impact of genes and nutrition on cardiovascular health. The ANN architecture and the module for personalized advice provision communicate through web-services technology and have been integrated into a web-based platform [43].

Future work includes the use of MDR method [44] in order to reveal certain main effects or interactions within the factors selected here, and construct rules that describe these. Furthermore, we intend to use the hybrid ANN methods presented here in the analysis of other multifactorial CVD risk factors, e.g. hypertension and the fasting level of the measurements of TG and LDL-C, and include other factors that describe a subject's lifestyle, e.g. related with physical activity and smoking. Furthermore, the optimization of ANNs used for the analysis of multifactorial disease traits is an open research area in terms of both ANN architecture and ANN training. Our final scope is to construct an ensemble of predictors that estimate the risk of developing CVD and develop methods able to assess the reduction of CVD risk after certain lifestyle interventions. The resulting panel of ANNs could be used to derive an overall predictive risk score to CVD based on genetics and lifestyle. Furthermore, since the overall risk is based on genes and lifestyle, and lifestyle is modifiable, it would be possible to create lifestyle scenarios where any high risk is reduced by means of "reverse engineering".

## Conclusions

In the current study, two ANN-based methods, namely the PDM-ANN and GA-ANN, an in house developed method for selecting the optimal set of input factors and architecture of an ANN, were used to study the multifactorial trait of obesity on the basis of a dataset of almost 2300 people. Associations beneath obesity, used here as an example of CVD risk factor, were searched within an set of 63 factors describing subjects' gender, genes and nutrition habits. Both methods concluded to parsimonious subsets of the original set of factors that affect BMI, a popular human weight measurement, and constructed appropriate predictive models for two BMI related classes. The most optimal set of factors was yielded by GA-ANN when a dimensionality threshold was applied during the stochastic process within the GA. The selected factors included gender, six genetic variations and 18 nutrition related variables and fed ANNs characterized by a promising generalization ability. The current work showed the importance of ANNs and their hybrids for the parallel processing of lifestyle and genetic data towards the analysis of modern disease related multifactorial traits.

## Authors' contributions

IV and SM designed the analysis, wrote the source codes, and drafted the manuscript. KG and KN provided advice on the project and revised the draft manuscript. All authors read and approved the final document.
